# Supplementary material for: Water deficit response in nodulated soybean roots: a comprehensive transcriptome and translatome network analysis
Source: BMC Plant Biol. 2024 Jun 21;24:585. doi: 10.1186/s12870-024-05280-5 (PMC11191192; doi:10.1186/s12870-024-05280-5)
Supplement: Supplementary file 6 — Supplementary Material 6 [file 12870_2024_5280_MOESM6_ESM.docx]

**Legends of supplementary figures**

**Figure S1. Descriptive analysis of the RNA-seq data.** **(A)** Heatmap of all samples comprising three replicates of each combined treated sample (N+WR; NN+WW; N+WW; NN+WW) and RNA fractions (TOTAL and PAR). **(B)** Principal component analysis.

**Figure S2. Functional enrichment analysis of the co-expression modules most representatives of the differentially expressed genes (DEGs) in the different contrasts between treatments. (A)** M3. **(B)** M5. **(C)** M6. **(D)** M7. **(E)** M9. **(F)** M10. **(G)** M11. **(H)** M12. The top ten Gene Ontology Biological Process (GO-BP) terms are shown for each module (y-axis).

**Figure S3. Water deficit assay imposition and monitoring.**

**(A)** Substrate water retention curves of WW plants comprising the two nodulation conditions (N and NN) during the water deficit assay (up to seven days). Stomatal conductance measurements (g_WS_) are shown for both N and NN plants, outlining the range of g_WS_ values (223-367 mmol m^-2^ s^-1^ and 118-337 mmol m^-2^ s^-1^ for N and NN plants, respectively) that the WW plants had during the water deficit assay. **(B)** Substrate water retention curves and stomatal conductance measurements (expressed as the % of the value obtained on day 0; %g_SW0_) of WR plants comprising the two nodulation conditions (N and NN) during the period of the water deficit assay (up to seven days). WW: well-watered plants; WR: water-restricted plants; N: nodulated plants; NN: non-nodulated plants; g_sw_: stomatal conductance. The dotted line in the g_SW0_ graphs indicates the % of g_sw_, with respect to day 0, at which the plants were considered to be stressed and were subsequently harvested. HP (%): amount of water in the substrate (sand:vermiculite, 1:1) expressed as a % of its dry weight. The dotted line indicates the HP (%) at which the substrate is at its field capacity (40% in the case of the substrate used in this assay).

**Figure S4. Identification of Venn diagram intersections.**
